# Current trends in access to treatment for hepatitis B in immigrants vs non-immigrants

**DOI:** 10.1093/gastro/goaa010

**Published:** 2020-03-27

**Authors:** Mireia Miquel, Albert Pardo, Montse Forné, Gemma Martínez-Alpin, Adrià Rodríguez-Castellano, Meritxell Casas, Mercè Rosinach, Mercè Roget, Blai Dalmau, Rocío Temiño, Joan Carlos Quer, Jordi Sanchez-Delgado, Jordi Ortiz, Mercedes Vergara

**Affiliations:** Liver Unit, Gastroenterology Department, Parc Taulí University Hospital, Institut d’Investigacio i Innovació Parc Taulí I3PT, Autonomous University of Barcelona, Sabadell, Spain; Centro de Investigación Biomédica en Red de Enfermedades Hepáticas y Digestivas (CIBERehd), Instituto de Salud Carlos III, Madrid, Spain; Gastroenterology Department, University Hospital Joan XXIII, Tarragona, Spain; Centro de Investigación Biomédica en Red de Enfermedades Hepáticas y Digestivas (CIBERehd), Instituto de Salud Carlos III, Madrid, Spain; Liver Unit, Gastroenterology Department, University Hospital Mútua Terrassa, Barcelona, Spain; Liver Unit, Gastroenterology Department, Consorci Sanitari de Terrassa, Terrassa, Spain; Gastroenterology Department, University Hospital Joan XXIII, Tarragona, Spain; Liver Unit, Gastroenterology Department, Parc Taulí University Hospital, Institut d’Investigacio i Innovació Parc Taulí I3PT, Autonomous University of Barcelona, Sabadell, Spain; Liver Unit, Gastroenterology Department, University Hospital Mútua Terrassa, Barcelona, Spain; Liver Unit, Gastroenterology Department, Consorci Sanitari de Terrassa, Terrassa, Spain; Liver Unit, Gastroenterology Department, Parc Taulí University Hospital, Institut d’Investigacio i Innovació Parc Taulí I3PT, Autonomous University of Barcelona, Sabadell, Spain; Liver Unit, Gastroenterology Department, University Hospital Mútua Terrassa, Barcelona, Spain; Gastroenterology Department, University Hospital Joan XXIII, Tarragona, Spain; Liver Unit, Gastroenterology Department, Parc Taulí University Hospital, Institut d’Investigacio i Innovació Parc Taulí I3PT, Autonomous University of Barcelona, Sabadell, Spain; Centro de Investigación Biomédica en Red de Enfermedades Hepáticas y Digestivas (CIBERehd), Instituto de Salud Carlos III, Madrid, Spain; Liver Unit, Gastroenterology Department, Consorci Sanitari de Terrassa, Terrassa, Spain; Liver Unit, Gastroenterology Department, Parc Taulí University Hospital, Institut d’Investigacio i Innovació Parc Taulí I3PT, Autonomous University of Barcelona, Sabadell, Spain; Centro de Investigación Biomédica en Red de Enfermedades Hepáticas y Digestivas (CIBERehd), Instituto de Salud Carlos III, Madrid, Spain

**Keywords:** hepatitis B, prevalence, immigration, access to treatment

## Abstract

**Background:**

Universal vaccination for hepatitis B virus (HBV) and migratory movements have changed the demographic characteristics of this disease in Spain and in Europe. Therefore, we evaluated the characteristics of the disease and the possible differences according to origin (immigrants vs non-immigrants) and access to treatment.

**Methods:**

This is a multicenter cross-sectional study (June 2014 to May 2015) in which outpatients with a positive HBsAg were seen and followed in four Hepatology units. Demographic and clinical data and indication and access to treatment were collected in two different regions of Catalonia (Spain) where there are no barriers to treatment due to a comprehensive coverage under the National Health System.

**Results:**

A total of 951 patients were evaluated (48.1% men). Of these, 46.6% were immigrants (58.7% of them were born in Africa) and were significantly younger compared to non-immigrants. The proportions of patients with alcohol consumption, being overweight, and other indicators of metabolic co-morbidities were significantly higher in non-immigrants. Among the 937 patients receiving HBeAg examination, 91.7% were HBeAg-negative. Chronic HBeAg-positive infection was significantly higher in immigrants (3.9% vs 0.6%, *P *=* *0.001) and chronic HBeAg-negative hepatitis was higher non-immigrants (31.7% vs 21.4%, *P *<* *0.001). Not only was the proportion of patients who met treatment criteria significantly higher among non-immigrants (38.4% vs 29.2%, *P *=* *0.003), but also the proportion of those with indication of effectively receiving therapy at the time of data collection (83.2% vs 57.8 %, *P *<* *0.001).

**Conclusions:**

The immigrant population with HBV is younger and has a lower prevalence of metabolic co-morbidities and a higher frequency of chronic HBeAg infection. Despite having access to care and an indication for treatment, some do not get adequately treated due to several factors including local adaptation that precludes access to treatment.

## Introduction

Two circumstances in recent decades have played an important role in the epidemiology of hepatitis B virus (HBV) infection in Europe. The first is the implementation of universal vaccination programs with a significant decrease in its prevalence [1, 2]. In a recent population study,<0.5% of HBsAg carriers were observed [3]. According to these data, Spain (which several years ago had been considered an area of intermediate prevalence [4]) is now a low prevalence region [5]. The second event is related to the significant flow of immigrants who currently represent 12% of the population [6]. Given that a significant proportion of these people come from areas of high prevalence of HBV, it has been considered that this new scenario could explain an attenuated decline in the infection rate in the general population [7, 8]. Presently, nearly half of the patients infected with HBV in Spain are immigrants [3]. There are scarce data on the impact of immigrants with HBV in Spain and Europe [9–13]. There are presumably some differences between the non-immigrant and immigrant population (such as the demographic characteristics, the evolutionary phase of the disease, and even cultural aspects) that may have significant implications in the diagnosis, follow-up, and management of the disease. Therefore, the main objective of this study was to analyse the characteristics of immigrant and non-immigrant patients infected with HBV monitored in four specialized Hepatology units. More specifically, we evaluated the differences including access to treatment between immigrant and non-immigrant populations in two large metropolitan areas in Spain where there are no expected barriers to treatment due to the comprehensive health coverage under the National Health System (NHS).

## Materials and methods

### Study design

**Figure 1.**
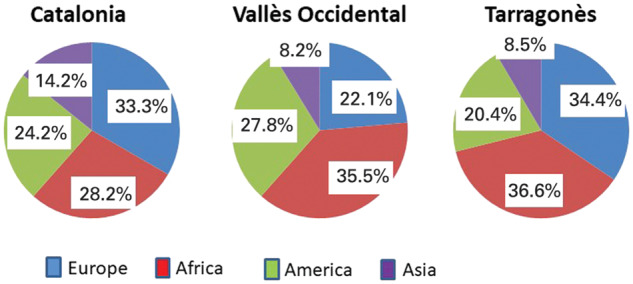
Origin of the immigrant population (Source: IDESCAT.CAT).

This was an observational multicenter cross-sectional study in four major reference hospitals in Catalunya, Spain (H. Parc Taulí, H. Consorci Sanitari de Terrassa, H. Mútua de Terrassa and Hospital Joan XXIII). The first three serve a population of around 900,000 inhabitants, of whom 9.68% are of immigrant origin, and the fourth (H. Joan XXIII) serves a population of about 250,000 inhabitants and a proportion of immigrants of 16.06% (Figure 1) [6]. All have protocols for the intake of immigrants that include the systematic determination of HBV serology as well as referral circuits between primary and specialized care for hepatitis B. The study was approved by the Clinical Research Ethics Committee of each center and was registered in the clinical trial database (NCT: 02548325). The information was collected via electronic medical records. The identity of patients was maintained confidential for all purposes, complying with the Organic Law of 15/1999, on the Protection of Personal Data. The principal investigator, in order to avoid the repeated inclusion of a same case in two of the participating centers, reviewed the database. If a duplication was detected, the record with the latest date was excluded.

### Study population

All patients with a positive HBsAg were evaluated in the Liver Clinic of each participating hospital between 1 June 2014 and 31 May 2015. We excluded those <18 years of age; those co-infected with HIV, hepatitis C, or delta hepatitis; and those in which the infection was acute. All those patients who were born in Spain (regardless of their family ethnic origin) were considered native cases (non-immigrant) and those who were born abroad as immigrants.

### Variables

The following clinical-variables data were collected: sex, date, and country of birth; the level of education (without studies, primary, high school, and university); and the presence of the usual epidemiological risk factors in relation to the transmission of hepatitis B (transfusion of blood products, intravenous drug use, tattoos or piercings, infected first-degree relatives, and high-risk sexual relationships). Likewise, the history of alcohol consumption (high-risk consumption was defined as that >30 grams per day) and the presence of other co-morbidities (diabetes mellitus, hypertension, dyslipidemia, and overweight) were recorded.

The following laboratory data were collected (that closest to the date of inclusion): complete liver biochemistry, serological markers of HBV (HBsAg, HBeAg, antiHBc, antiHBs, and antiHBe) and HBV viral load. In all the centers, the quantification of the HBV DNA was carried out by real-time polymerase chain reaction with a measurement interval between 20 and 1,700,000,000 IU/mL. The criteria recently defined by the European Association for the Study of the Liver (EASL) were used to characterize patients into four groups with respect to the evolutionary phase of the infection: (i) chronic HBeAg-positive infection, (ii) chronic HBeAg-positive hepatitis, (iii) chronic HBeAg-negative infection and (iv) chronic HBeAg-negative hepatitis. We evaluated whether patients met criteria for therapy according to EASL guidelines [14]. Likewise, data on whether the patients were effectively receiving antiviral treatment or not according to these recommendations were collected. In cases in which liver biopsy or elastography was performed, the METAVIR classification for fibrosis and the liver-stiffness value in kPa was recorded (a threshold of 7.5 kPa was considered to establish the suspicion of significant fibrosis) [15].

### Statistical analysis

Continuous variables are expressed as mean with standard deviation or median with interquartile range (IQR) and the qualitative variables as percentages with a confidence interval of 95%. The continuous variables were compared with the Student’s *t*-test in the variables of normal distribution. Non-parametric values were evaluated with the McNemar test. The categorical variables are compared with chi-square test and the ordinal categorical variables with rank-sum test. To perform the statistical analysis, we used the SPSS program, version 21. A *P *<* *0.05 was considered significant.

## Results

A total of 998 patients with a positive HBsAg were included. Of these, 47 were excluded: 30 due to co-infection with another virus (13 HCV, 13 HDV, and 4 HIV), 12 due to acute HBV infection, and 5 due to age <18 years. Finally, 951 cases were analysed; 305 (32.1%) in H. Parc Taulí, 124 (13.0%) in Mútua de Terrassa, 124 (13.0%) in Consorci Sanitari de Terrassa, and 398 (41.9%) in H. Joan XXIII. Non-immigrant patients had a median follow-up of 5.17 years (IQR: 10.55) and immigrant patients of 3.25 years (IQR: 3.25) (*P *<* *0.001).

### Demographic characteristics

There were 508 (53.4%) non-immigrant and 443 (46.6%) immigrant patients. Immigrants came from: Africa 260 (58.7%), other parts of Europe 88 (19.9%) (mainly from East-Europe: Romania 48 patients, Bulgaria 10 patients), Asia 69 (15.6%), and Latin America 26 (5.9%). There were no diffrences among centers (Figure 1). Immigrants were significantly younger than non-immigrants (36 ± 10 vs 54 ± 12 years, *P *<* *0.001) without differences in distribution by gender. Immigrants had significantly higher education level than non-immigrants (*P *=* *0.036) (Table 1).

**Table 1.** Demographic and clinical characteristics of both populations

Characteristic	Global (*n* = 951)	Non-immigrant (*n* = 508)	Immigrant (*n* = 443)	*P*-value
Age, years	46 ± 14	54 ± 12	36 ± 10	<0.001
Male gender	457 (48.1)	249 (49.0)	208 (47.0)	>0.05
Education level	*n* = 397	*n* = 235	*n* = 162	0.036
Without studies	48 (12.1)	27 (11.5)	21 (13.0)	
Primary	195 (49.1)	131 (55.7)	64 (39.5)	
High school	113 (28.5)	55 (23.4)	58 (35.8)	
University studies	41 (10.3)	22 (9.4)	19 (11.7)	
Transmission routes	*n* = 151	*n* = 84	*n* = 67	
First-degree family/vertical	42 (27.8)	8 (9.5)	34 (50.7)	<0.001
Post-transfusion	36 (23.8)	29 (34.5)	7 (10.4)	0.005
Piercings/tattoos	47 (31.1)	31 (36.9)	16 (23.8)	>0.05
Intravenous drug user	3 (2.0)	2 (2.4)	1 (1.5)	>0.05
Sexual risk behavior	23 (15.2)	14 (16.6)	9 (13.5)	>0.05
Alcohol-consumption history	85 (15.8)	67 (21.0)	18 (8.3)	<0.001
Overweight (BMI > 25 kg/m^2^)	498 (52.4)	314 (61.8)	184 (41.5)	<0.001
Co-morbidities	*n* = 553	*n* = 323	*n* = 230	
Arterial hypertension	73 (13.2)	60 (18.6)	13 (5.7)	<0.001
Dyslipidemia	78 (14.1)	66 (20.4)	12 (5.2)	<0.001
Diabetes mellitus	37 (6.7)	30 (9.3)	7 (3.0)	0.003

Values are presented as mean ± standard deviation or *n* (%).

BMI, body mass index.

### Epidemiological risk factors

The history of infected first-degree relatives was significantly higher among immigrants (15.9% vs 2.7%, *P *<* *0.001), while a history of transfusion was more frequent among non-immigrants (9.3% vs 3.2%, *P *=* *0.005). No significant differences were found regarding a history of tattoos, piercing, current or previous drug use, and high-risk sexual contacts. The reported alcohol consumption was significantly higher among non-immigrants (21.0% vs 8.3%, *P *<* *0.001), as well as being overweight and other indicators of metabolic co-morbidity (hypertension, dyslipidemia, and diabetes mellitus) (Table 1).

### Characteristics of the infection

**Figure 2.**
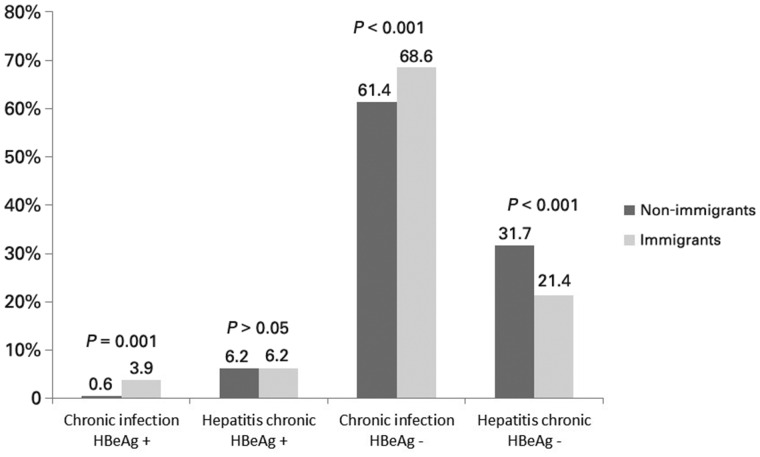
Disease liver stage in non-immigrants and immigrants.

HBeAg was examined in 937 patients (98.5%). Of the 859 patients (91.7%) with a negative HBeAg, 607 (64.8%) were classified as ‘chronic HBeAg-negative infection’ (formerly called inactive carriers) and 252 (26.9%) as ‘chronic HBeAg-negative hepatitis’. Of the 78 patients (8.3%) with a positive HBeAg, 58 (6.2%) were classified as ‘chronic HBeAg-positive hepatitis’ and only 20 (2.1%) as ‘HBeAg-positive chronic infection’ (formerly called immunotolerant). The proportion of cases in the phase of ‘chronic HBeAg-positive infection’ was significantly higher among immigrant patients (3.9% vs 0.6%, *P *=* *0.001), while the frequency of cases in the ‘chronic HBeAg-negative hepatitis’ was higher among non-immigrants (31.7% vs 21.4, *P *<* *0.001) (Figure 2).

In 709 patients (75.7%), transient elastography was performed. Although the mean value (kPa) of liver stiffness was higher in immigrant patients (7.0 ± 4.7 vs 6.3 ± 3.6, *P *=* *0.02), the proportion in which the value was considered suspicious for significant fibrosis (>7.5 kPa) was higher among non-immigrants but without significance (23.9% vs 18.9%, *P *=* *0.1).

Liver biopsy was performed in 188 patients (19.8%) and this proportion was significantly lower among immigrants vs non-immigrants (16% vs 24%, *P *=* *0.002). There were no statistically significant differences between both groups regarding the distribution between the different degrees of fibrosis observed in the biopsy according to the METAVIR classification (Table 2).

**Table 2.** Characteristics of the disease and treatment

Characteristic	Global (*n* = 937)	Non-immigrant (*n* = 498)	Immigrant (*n* = 439)	*P*-value
HBeAg-positive	78 (8.3)	34 (6.8)	44 (10.0)	>0.05
Transient elastography	*n* = 709	*n* = 376	*n* = 333	
LSM, kPa	6.5 ± 4.1	7.0 ± 4.7	6.3 ± 3.6	0.020
LSM > 7.5 kPa	153 (21.6)	90 (23.9)	63 (18.9)	>0.05
Fibrosis degree by liver biopsy	*n* = 188	*n* = 119	*n* = 69	>0.05
F0	77 (41.0)	49 (41.1)	28 (40.6)	
F1	28 (14.9)	18 (15.1)	10 (14.5)	
F2	26 (13.8)	15 (12.6)	11 (15.9)	
F3	48 (25.5)	33 (27.7)	15 (21.7)	
F4	8 (4.3)	4 (3.4)	4 (5.8)	
Treatment indication	319 (34.0)	191 (38.4)	128 (29.2)	0.003
Under treatment	233 (73.0)	159 (83.2)	74 (57.8)	<0.001

Values are presented as mean ± standard deviation or *n* (%).

LSM, liver-stiffness measurement.

### Treatment indication

**Figure 3.**
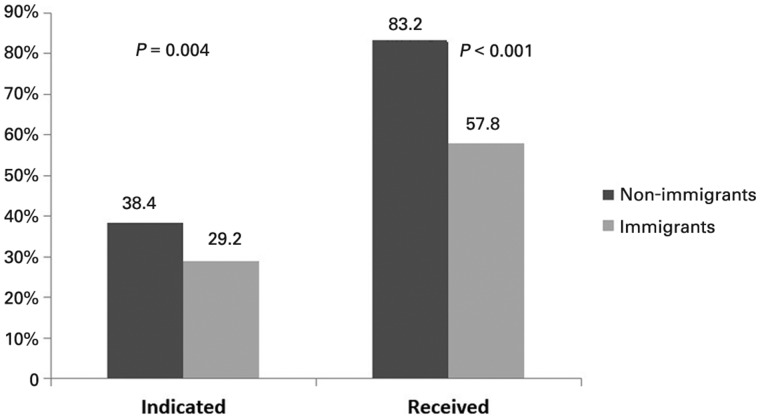
Differences in treatment indicated and treatment effectively received between non-immigrants and immigrants. The two columns on the left show the percentage of patients with treatment criteria. The two on the right show the percentage of patients who receive treatment of those who have an indication.

According to EASL guidelines, 319 patients (34.0%) fulfilled at least one of the criteria for treatment. The proportion was significantly higher in non-immigrants (38.4% vs 29.2%, *P *=* *0.004). Likewise, the proportion of patients with a treatment indication who, in fact, were receiving any type of therapy at the time of inclusion in the study was higher among non-immigrants compared to immigrants (83.2% vs 57.8%, *P *<* *0.001) (Figure 3).

## Discussion

The current study demonstrates that immigrants with chronic HBV who are candidates for antiviral therapy have low access to treatments compared to non-immigrants. In addition, the results from this analysis confirm that immigrants infected with HBV represent a sizable proportion of patients in urban areas. These results are comparable to those observed in a recent study (conducted from a population perspective) which estimates that 55% of patients infected with HBV in a region of Spain are of immigrant origin [3]. This highlights the fact that the population prevalence of HBV infection in Spain, which is already below intermediate values, would be even lower if this demographic change had not occurred [7]. To our knowledge, there are no published studies addressing these discrepancies in Spain or Europe. The study also demonstrates that immigrant patients are younger due to a higher vertical transmission and most are immunotolerant. This fact is not surprising given that a large number of the immigrant population come from regions of high prevalence, where this route of transmission is more frequent. On the other hand, non-immigrants were older, had a horizontal transmission (i.e. transfusion history), and had other cofactors such as the consumption of alcohol and/or the presence of metabolic co-morbidity.

Although the study did not show large differences in relation to the phase of the infection (except for the higher prevalence of immunotolerants in immigrant patients) or in the stage of fibrosis evaluated by elastography and liver biopsy, it did identify a higher percentage with an indication for therapy in non-immigrants. A possible explanation for this is likely due to age and cofactors such as alcohol or being overweight. In addition, one of the aspects that we consider most relevant was that the proportion of immigrants that were not treated despite meeting criteria of the EASL recommendations [14]. Thus, 42% of the immigrant patients with an indication of treatment were not receiving it at the time of inclusion, whereas this only occurred in 17% of the non-immigrants. There are several reasons that could explain these inequalities. One is that the decision to treat takes into account the evolution of the disease. Since immigrants have short follow-up after they are first seen, this precludes many of them from getting adequate therapy. In a previous study carried out in one of the participating centers, it was confirmed that the loss of follow-up, which in many cases occurred before the end of the evaluation period and the indication for treatment, was significantly higher among immigrant patients [16]. Another reason is related to the problems with local adaptation, as well as family and work stability, which are often lower among the immigrant population and could be determining factors. This lower access to treatment in the immigrant population is not exclusive in this pathology [17–19]. In fact, there are data which demonstrate that the immigrant population, in general, has less access to the health system and therefore to specific treatments [20–23].

One of the strengths of this study is that it is a multicenter study, with percentages of immigration that may be representative of the territory and other areas of Europe. Thus, it provides data on access to treatment in accordance with the latest EASL recommendations. However, a limitation is that it is a cross-sectional study. Consequently, it was not possible to collect the data from all the patients lost from follow-up, which would allow determining whether this is one of the causes of the lower percentage of patients treated. Another limitation is the lack of a prospective follow-up that would allow us to delve into the causes of the lower rate of treatment.

In conclusion, patients of immigrant origin are younger, with a lower prevalence of metabolic co-morbidity and a higher frequency of HBeAg-positive infection. Although, in general, immigrants present in an evolutionary phase of the disease very similar to that of non-immigrants, a lower percentage of treated cases was observed in a country with a universal healthcare system. Although it would be necessary to have more data, these results suggest the need of additional efforts in order to improve adherence to follow-up in consultations in this particular group of patients.

## Authors’ contribution

M.M. and A.P. participated in the design, inclusion of patients, statistical analysis, and drafting of the manuscript. M.F., G.M.A., A.R.C., M.C., M Rosinach, M Roget, J.C.Q., and M.V. participated in the design, inclusion of patients, and critical revision of the manuscript. B.D., R.T., J.S.D., and J.O. participated in critical revision of the manuscript. All authors have read and approved the final manuscript.

## Funding

No funding has been received for this study.
